# *In-vivo* activation of vomeronasal neurons shows adaptive responses to pheromonal stimuli

**DOI:** 10.1038/s41598-018-26831-5

**Published:** 2018-05-31

**Authors:** Lucia Silvotti, Rosa Maria Cavaliere, Silvana Belletti, Roberto Tirindelli

**Affiliations:** Department of Medicine and Surgery, Neuroscience Unit, University of Parma, Via Volturno, 39, 43125 Parma, Italy

## Abstract

In most mammals, the vomeronasal system has a pivotal role in mediating socio-sexual behaviours. The vomeronasal organ senses pheromones through the activation of specific receptors. Pheromone binding to cognate receptors activates Ca-influx via the gating of a cation channel that generates membrane depolarisation. The *ex-vivo* activation of vomeronasal neurons (VSNs) by pheromonal stimuli has been largely investigated by electrophysiological and imaging techniques; however, few studies have been carried out to determine the physiological responses of VSNs, *in-vivo*. By tracking the phosphorylation of S6 ribosomal protein as a marker of neuronal activity, we show that S6 becomes phosphorylated (pS6) in mouse VSNs stimulated by intraspecific and heterospecific pheromonal cues. We observed that female scent induces pS6 immunoreactivity in the apical VSNs of male vomeronasal epithelium, whereas male cues stimulate S6 phosphorylation in both the basal and apical VSNs of females. We also show that this dimorphic pattern of pS6 immunoreactivity is reproduced when heterospecific stimuli are used. Moreover, we found that a consistent proportion of VSNs is activated by both heterospecific and intraspecific pheromones. Additionally, we have evidence of adaptive responses to S6 phosphorylation when stimulation with cues of the same and opposite sex and of different species is sustained.

## Introduction

Chemosensory perception is based on the detection of molecules that trigger physiological, reproductive and social responses including food search and avoidance, escape from predators, mating, aggressiveness and attractiveness. The vomeronasal organ of Jacobson (VNO) is an olfactory structure that is specifically responsible for converting chemical signals, pheromones, stemming from hetero- and conspecific animals, into neuroendocrine responses, thus modulating the social life of many species^1–3^. The mouse VNO is equipped with chemosensory neurons (VSNs) expressing two large superfamilies of pheromone receptors, namely type-I (V1Rs) and type-II (V2Rs) vomeronasal receptors, and a small family of formyl peptide receptors (FPRs) that are believed to detect pathogenic molecules emitted by sick animals^4–8^. The expression pattern of these receptor families is complementary as V1Rs and FPRs are preferentially expressed in the apical chemosensory neurons of the VNO, whereas V2Rs are in the basal neurons. V1Rs and FPR reportedly bind small molecules whereas V2Rs bind polypeptides^9–13^. All of these receptors are believed to be equally expressed in males and females, thus excluding a consistent sexual dimorphism of the VNO^13,14^. In contrast, differences between sexes primarily occur in the content of male and female pheromonal secretions^15–20^. VSN responses initiate with the binding of pheromones to their cognate receptors and the resulting stimulation of two specific G-proteins, Gαi2 (co-expressing with V1Rs) and Gαo (co-expressing with V2Rs). This results in activation of the phospholipase-C signalling cascade with the production of diacylglycerol, which causes opening of the transient receptor potential channel (TrpC2), an increase in intracellular calcium and the generation of action potentials^19–22^. The electrical signal is thus propagated to dedicated central areas of the nervous system, specifically committed to elicit neuroendocrine and behavioural responses^1,23^. Hence, whereas the signal transduction mechanisms underlying desensitization to odour stimuli are quite well defined in the main olfactory epithelium^24^, the existence of adapting mechanisms to pheromonal stimuli that are physiologically relevant are still controversial in the VNO^17,20,25,26^. On the one hand, adapting mechanisms would be disadvantageous to sustain a complete or prolonged behavioural response, while, on the other hand, if absent, they would endure VSNs with a continuous background stimulation elicited by self, sex and strain odours, thus limiting the range of signal detection and discrimination, *in vivo*. However, *in vivo*, VSN activity, upon pheromonal stimulation, is difficult to track by the immunological detection of the traditional neuronal markers c-Fos or Egr 1 because they are poorly expressed^27–29^, although an exhaustive study, using *in situ* hybridisation to detect the up-regulation of RNA expression of the *Egr 1* gene, has clarified many aspects of the pheromonal transduction^12^. In particular, this study focused on the association of vomeronasal receptors with cognate hetero- or intraspecific stimuli establishing the molecular basis of pheromonal information coding.

Few years ago, Knight and colleagues developed a method to determine *in-vivo* activation of neurons in the central nervous system^30^. They found that the S6 ribosomal protein is phosphorylated in response to a wide variety of stimuli; this phosphorylation is comparable to the up-regulation of immediate early gene expression, such as c-Fos and Egr 1. Following this study, Jiang and colleagues reported that S6 phosphorylation, *in-vivo*, occurs in the olfactory neurons in response to the binding of specific odorants to their cognate receptors^31^. While this manuscript was under revision, Tsunoda and colleagues reported the activation of a subpopulation of rat and mouse vomeronasal neurons by S6 phosphorylation in response to rat tears^32^. This suggests that pS6 is also a reliable marker to determine neuronal activation in the peripheral olfactory and sub-olfactory systems.

Based on these previous observations, we have tracked VSN activity via the immunological detection of the phosphorylated S6 ribosomal subunit following *in-vivo* stimulation with different pheromonal cues. We confirm that this method is time saving, reliable and sensitive for any given pheromonal stimulus. Here, we show that phosphorylation of the S6 ribosomal subunit markedly and rapidly increases in the VSNs following exposure to intraspecific and heterospecific pheromones. Interestingly, complete dephosphorylation of pS6 occurs if stimulation with intraspecific and heterospecific cues is sustained, leaving VSN unable to respond to the same stimuli, via S6 rephosphorylation, for several hours. Thus, this work opens the question as to whether long-term filtering mechanisms of pheromonal stimuli exist in the VNO.

## Results

### Optimisation of the experimental protocol

In preliminary experiments to assess the temporal profile of pS6 expression in the VNO upon contact to pheromonal stimulation, CD1 male mice were introduced in a cage containing bedding mix of male and female Balb/c mice or to clean bedding for an hour and then sacrificed 0′, 60′ and 120′ after removal of the stimulus (see Material and methods for the experimental procedures). Our results indicated that pS6-immunoreactive cells dramatically increased compared to the control (clean bedding) in the VNO immediately after 1 hr exposure to the stimulus (0′) (F = 11.689, p < 0.001) and the immunological signal remained unchanged for the remaining 120′ (Supplementary Fig. S1). Next, we evaluated the shortest time in contact with the pheromonal cues that was required to elicit a significant increase in pS6 immunoreactivity. Therefore, we exposed male mice to the bedding mix for 10′ and then transferred them to a cage with clean bedding for 50′ prior to sacrifice. We found that this short exposure time to pheromones was sufficient to elicit an almost complete response in the VNO, which was comparable to that observed in animals exposed to the same stimuli for 1 hr (Supplementary Fig. S1).

Although the large number of pS6 positive cells detected in the VNO after stimulation with soiled bedding was compatible with activated chemosensory neurons, we employed a gene-targeted mouse strain (OMP-GFP) in which the expression of GFP (green fluorescent protein) is driven by the promoter of the *omp* (olfactory marker protein) gene, to visualise pS6 expression in mature VSNs. Our results indicated that about 90% of pS6 positive neurons also expressed GFP (Supplementary Fig. S2); therefore, neuronal activation was strictly related to phosphorylation of the ribosomal protein S6. Thus, in light of this preliminary analysis, we standardised the protocol so that all experiments were performed by exposing wild type CD1 mice to pheromonal scents for a fixed time of 60′ prior to sacrifice unless otherwise indicated.

### S6 phosphorylation following stimulation with intraspecific pheromonal cues

**Figure 1:**
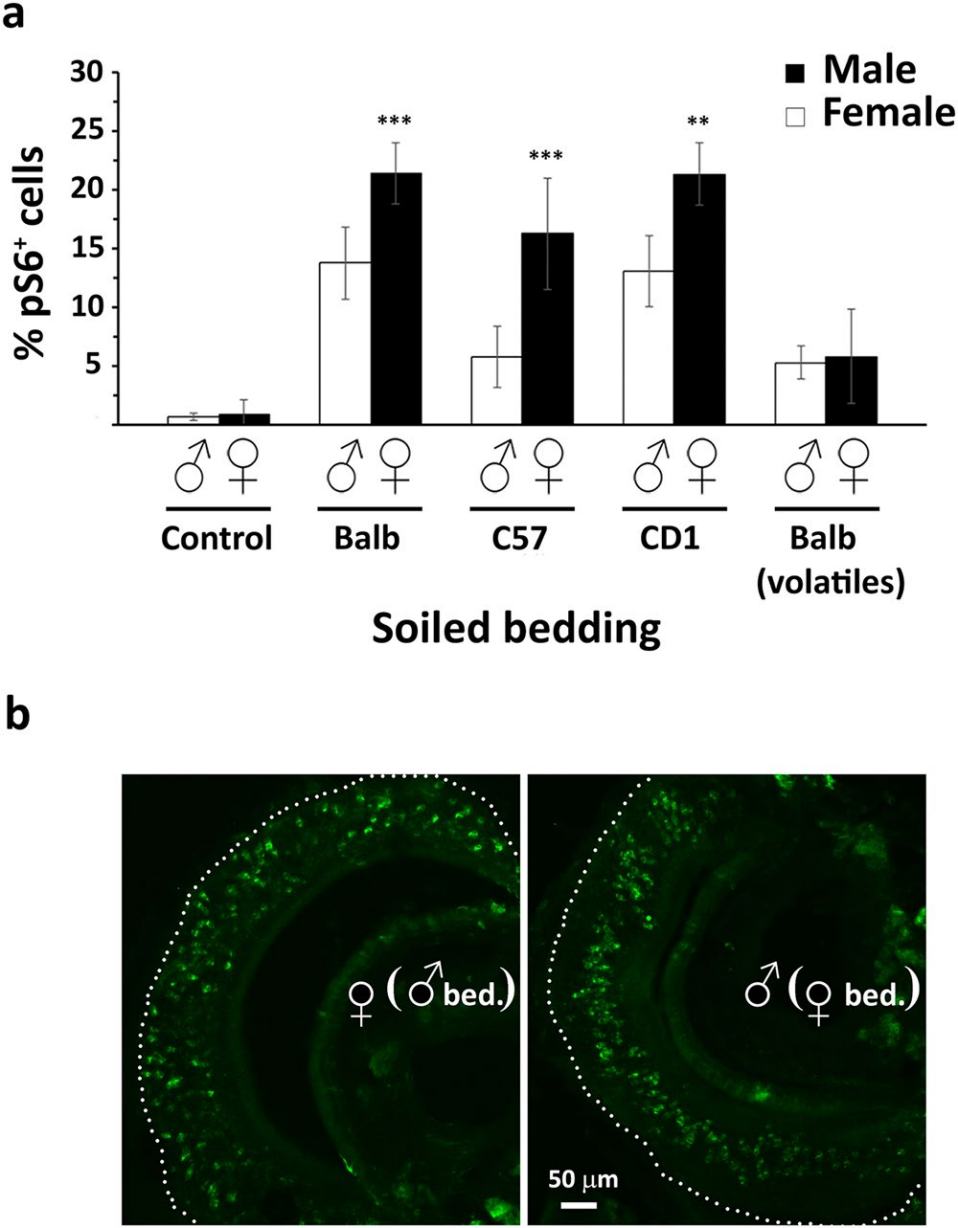
Expression of pS6 in mice stimulated with intraspecific soiled bedding. **(a)** S6 phosphorylation in male and female mice exposed to bedding from different sexes and strains or exposed to clean bedding (control). In all mouse strains, female cues are more effective than male cues in activating VSNs. Male C57/Bl6 bedding is less effective than CD1 and Balb/c bedding in activating female VNO. The difference is abolished when mice are not permitted to have physical contact with bedding (n = 7 for each group, means ± s.d.; ***p < 0.001; **p < 0.01). **(b)** pS6 immunoreactivity of VNO sections of male and female mice exposed to female and male soiled bedding, respectively. Female stimuli induced pS6 phosphorylation in neurons of the upper layer of male VNO. In contrast, pS6 immunoreactivity is scattered throughout the VNO in females stimulated with male bedding.

We also wanted to investigate the changes in pS6 expression in the VNO of CD1 females and males exposed to soiled bedding collected from mice of the same or opposite sex. The first observation was that male VNO responded to the opposite sex bedding with a higher density of pS6-positive cells than female VNO to male bedding. This was true when bedding from Balb/c (F = 1.265, p = 0.001), C57/Bl6 (F = 0.91, p = 0.001) and CD1 (F = 0.084, p = 0.002) strains was employed as stimuli (Fig. 1a). In general, we noticed that the pheromonal cues present in the bedding of C57/Bl6 mice were less stimulatory than those contained in the bedding of other mouse strains. Specifically, in males, C57/Bl6 female bedding induced a lower increase of pS6 immunoreactivity than the bedding of Balb/c and CD1 female (Balb/c, F = 1.27, p < 0.001; CD1, F = 0.029, p = 0.002) (Fig. 1a). Moreover, we found a different distribution of the pS6-positive VSNs since female stimuli preferentially activated apical neurons of the male VNO, whereas male bedding stimulated female VSNs of both neuronal layers (Fig. 1b). To further confirm this observation, we performed double label immunohistochemistry with an antibody, which recognises all V2R-expressing neurons (panC) that are known to be located basally in the VNO^33^. Our data showed that, for both female and male stimuli, only a very low percentage of V2R-expressing neurons co-expressed with pS6; however, male cues proportionally activated more V2R-expressing neurons in female VNO (13%) than the opposite condition (6%) (F = 1.134; p = 0.001) (Supplementary Fig. S3). Together, these results point out that female body scent displays a more complex pheromonal repertoire than males and that significant differences may occur in the pheromonal blend of various strains. Thus, this led us to scrutinise the chemical nature of the active pheromones contained in bedding of the two sexes. Therefore, we carried out experiments where CD1 male and female mice were prevented from having physical contact with beddings of the opposite sex, allowing the detection of volatile molecules only. Our data showed a significant decrease in the number and intensity of pS6-expressing neurons in both female and male VNO (female, F = 12.317, p < 0.001; males, F = 2.582, p < 0.001) (Fig. 1a). Of particular note, we found no difference in the number of pS6-positive cells between females and males (Fig. 1a). As expected, since V2R-expressing neurons are believed to sense non-volatile pheromones, the percentage of pS6-positive neurons expressing V2Rs declined in these conditions (Supplementary Fig. S3). This suggests that non-volatile molecules strongly contribute to the high pheromone content of the female scent.

### Prolonged stimuli do not sustain S6 phosphorylation

**Figure 2:**
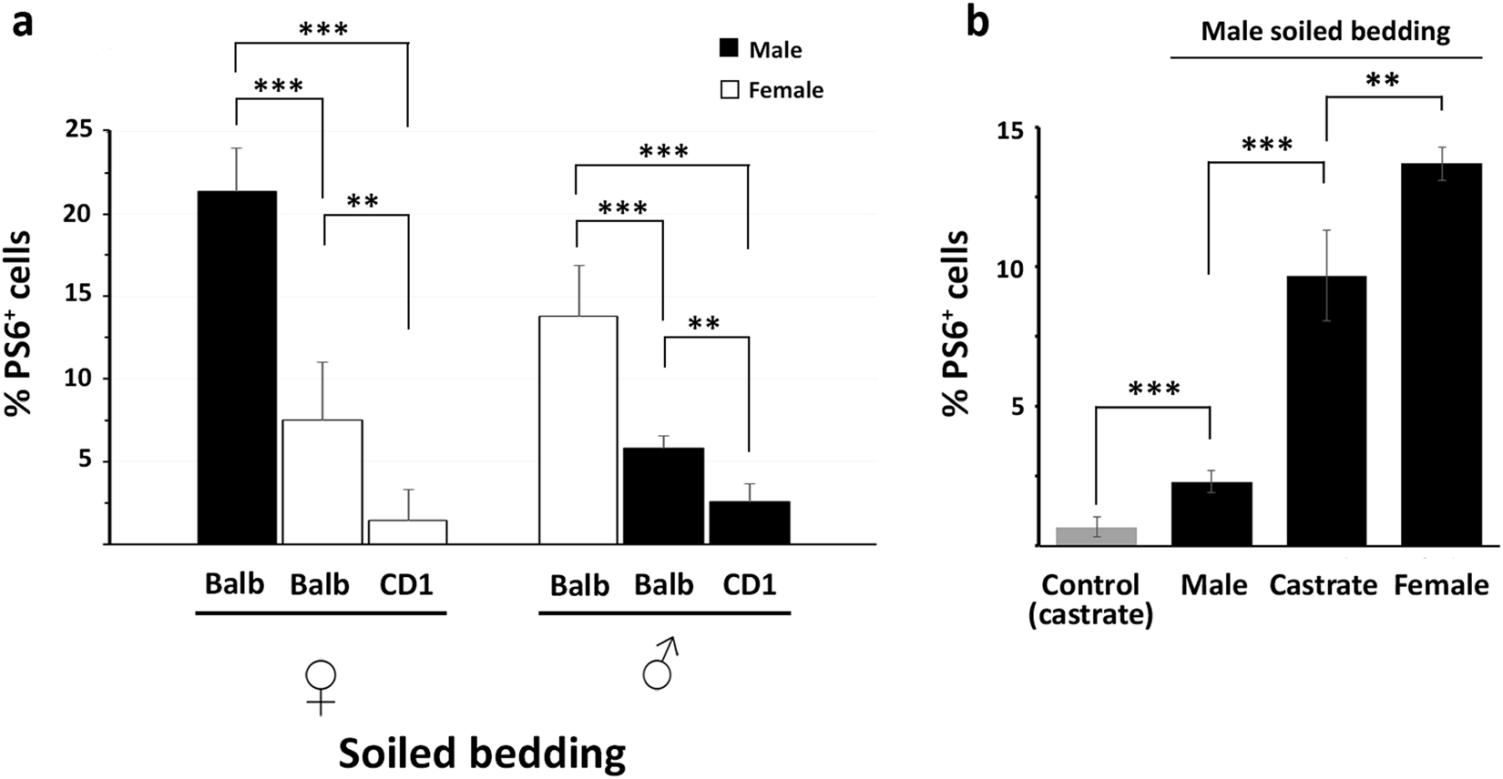
The VNO of male and female mice shows adaptation to same sex stimuli. **(a)** Compared to opposite sex stimuli, the percentage of pS6-positive cells decreases when animals are exposed to soiled bedding of the same sex. This adaptive response is stronger when stimuli of the same strain are employed. Bars 1 and 4 are from Fig. 1 (**b**) Male, female and castrated male mice are exposed to male mouse soiled bedding. Castrated males, compared to intact males, show a reduced adaptive response to the stimulus. Controls are castrated mice exposed to clean soiled bedding (n = 7 for each group, means ± s.d.; ***p < 0.001; **p < 0.01).

When female or male mice were exposed to bedding of the same sex, we observed a lower VNO activation than that generated by a stimulus of the opposite sex (Balb/c female bedding, F = 0.538, p < 0.001; Balb/c male bedding, F = 29.265, p < 0.001) (Fig. 2). Moreover, the sensitivity of the VNO to stimuli of the same sex further decreased when odour cues originated from bedding of the same strain of the experimental animal (CD1) (female CD1 bedding, F = 2.534, p = 0.002; male CD1 bedding, F = 0.690, p = 0.001) (Fig. 2a). In addition, the spatial distribution of the activated neurons by the same sex stimuli in the VNO was not identical in females and males. In fact, in males, pS6 immunoreactivity was more restricted to basal neurons of the VNO, whereas, in females, pS6-positive cells were circumscribed to the apical layer (Supplementary Fig. S4). Accordingly, a higher percentage of pS6-positive neurons in males than in females also expressed the basally located V2Rs (F = 0.397, p < 0.001) (Supplementary Fig. S3). Overall, these results suggest the existence of adaptive mechanisms to self (and strain) odours in VSNs. This was also confirmed in experiments on castrated male mice, which do not produce masculine pheromones. When these mice were exposed to bedding of intact male mice, the sensitivity of VSNs was strongly increased compared to intact males, indicating a loss of adaptation to same sex stimuli (F = 2.14, p < 0.001) (Fig. 2b).

**Figure 3:**
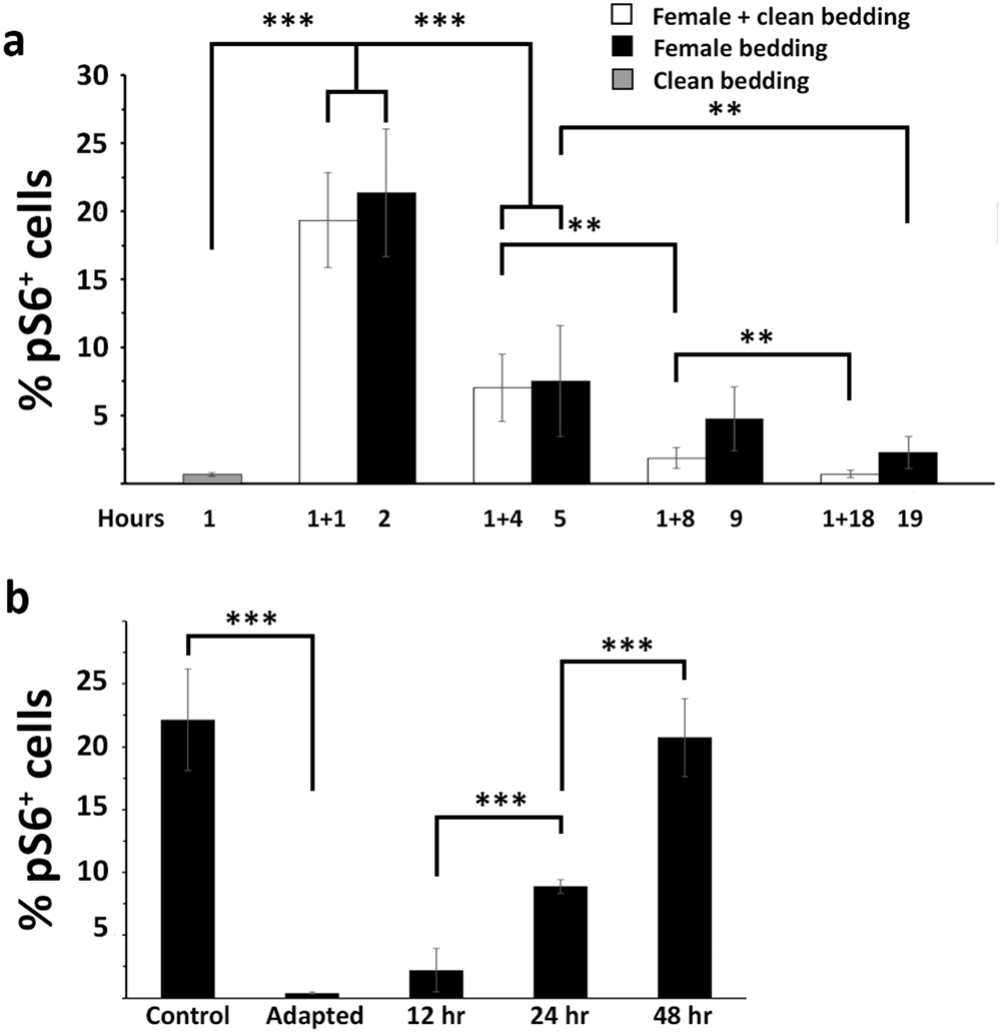
Time course of the adaptive response to opposite sex stimuli. **(a)** Male mice are exposed to Balb/c female bedding for increasing time intervals and the percentage of pS6-positive neurons is measured (black bars). Ninety percent of the adaptive response takes place in the first 19 hours. White bars depict the decay profile of pS6 immunoreactivity after exposing mice to bedding for 1 hr and to clean bedding for the remaining time before sacrifice. Control (grey bar) corresponds to the exposure of mice to clean bedding for 1 hr prior to sacrifice (n = 7 for each group, means ± s.d.). **(b)** Male mice are fully adapted to Balb/c female bedding for 3 days and then moved to a clean cage for the indicated time before stimulation for 1 hr with the same bedding as employed for the adaptation. As a control, isolated CD1 mice are exposed to the same Balb/c female bedding for 1 hr before sacrifice. The VNO is fully resensitized after 48 hrs.

To better investigate this issue and assess whether an adaptive process also occurs with opposite sex stimuli, we exposed male mice to female bedding for different time intervals prior to sacrifice (Fig. 3a). As a control, we traced the temporal profile of pS6 expression, by stimulating males with female bedding for 1 hr and then sacrificing them at the same intervals as for the experimental animals, but after isolation in a cage with clean bedding for the remaining time. We reasoned, in fact, that if the adaptation of VSNs to pheromonal stimuli did occur, the temporal profile of pS6 expression in experimental animals would show a decline over time. Indeed, pS6 immunoreactivity in experimental animals did not significantly differ from that of controls after 2 hr exposure to bedding; however, in both cases, immunoreactivity significantly decreased by 70% after 5 hours. From here onwards, in the experimental animals, adaptation seemed to proceed slightly slower (but not significantly) than the natural decay of the immunoreactivity that reached the background level after 19 hours from the first contact with the stimulus (Fig. 3a). Finally, we measured the amount of time required to recover from adaptation, by isolating female scent-adapted males in a cage containing clean bedding for increasing time intervals prior to restimulation with the same female bedding that was used for adaptation. Our results indicated that the S6 protein could be fully rephosphorylated upon stimulation only after 48 hrs from the last contact with the same stimulus (Fig. 3b). Overall, these results suggest that VSNs undergo adaptation following sustained stimulation with pheromonal cues of the same and opposite sex and that the process of resensitization of the VSNs requires several hours before it is fully accomplished.

### Heterospecific cues stimulate S6 phosphorylation and partially desensitize VSNs to intraspecific stimuli

**Figure 4:**
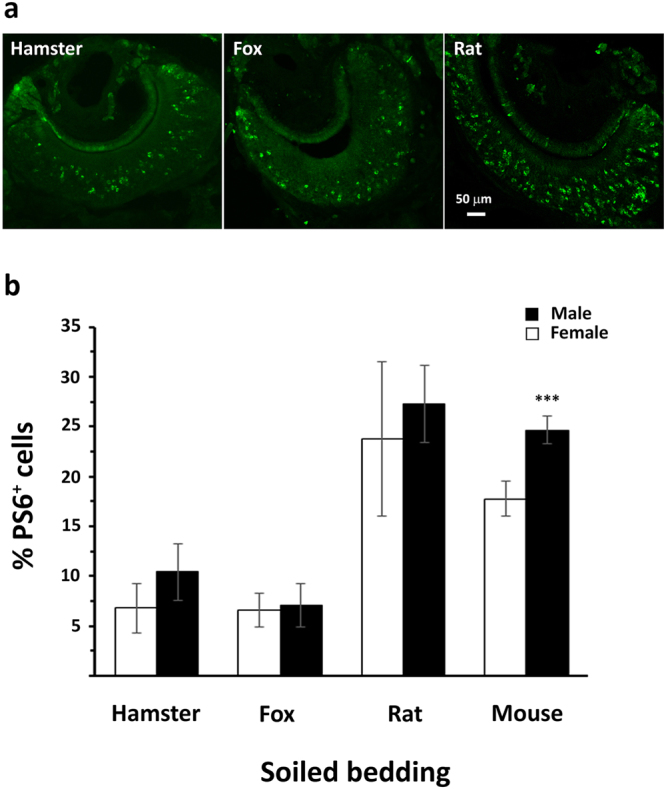
Female and male VNO responses to heterospecific stimuli. **(a)** pS6 immunoreactivity in the VNO of male mice exposed to a bedding mix (female and male) or urine (fox) of heterospecific animals. **(b)** Mice are exposed to a bedding mix (female and male) or urine (fox) of heterospecific animals and the percentage of pS6 positive cells are measured. No significant sex difference in detecting heterospecific cues is observed. In contrast, pS6 positive cells are higher in males exposed to mouse bedding mix than in females (n = 7 for each group; means ± s.d.; ***p < 0.001).

We next assayed pS6 immunoreactivity in the VNO of mice exposed to heterospecific stimuli of both predator and non-predator species (Fig. 4). In this experiment, we used a stimulus consisting of a bedding mix of both sexes of hamster and rat and of a commercial sample of fox urine sprayed onto clean bedding. As a control, we employed a bedding mix of female and male mice. Except for the control (mouse bedding mix), which showed a higher pS6 immunoreactivity in males than females VNO, as expected, we could not identify differences in the number of pS6-positive neurons between sexes when heterospecific stimuli were administered to mice. We also tested a commercial sample of bobcat urine, but this was unable to stimulate mouse VNO (data not shown). Although rat bedding was found to be a powerful stimulus, activating up to 30% of the total VNO neurons, we observed that the percentage of pS6-positive neurons expressing V2Rs was relatively low (Supplementary Fig. S3). Interestingly, we found that female rat bedding preferentially activated apical VSNs, whereas male rat cues stimulated VSNs in both layers (Supplementary Fig. S5). Since this spatial distribution was identical for mouse stimuli (Fig. 1), we were prompted to investigate to what degree mouse and rat scent activated the same vomeronasal neurons. To accomplish that, male mice were fully adapted to female mouse bedding for 48 hrs and then stimulated with female rat bedding for 60′ before sacrifice. Our results indicated that after full adaptation, pS6 immunoreactivity significantly decreased by about 33% (F = 4.88; p = 0.001) compared to non-adapted mice exposed to the same female rat stimuli (Supplementary Fig. S6). These data suggest that one third of female mouse and rat pheromones share the same VNO receptors.

Since it was recently reported that VSNs expressing the RNA of a specific group of V2R (subfamily D) exclusively responded to heterospecific stimuli^12^, we stimulated male mice with rat, hamster and mouse bedding and counterstained pS6-positive neurons with an antibody recognising all members of subfamily-D V2Rs (panD)^34^. Unexpectedly, we could not observe a preferential co-expression of pS6 with subfamily-D V2Rs in response to heterospecific stimuli (Supplementary Fig. S7).

### S6 phosphorylation following interactive behaviours

**Figure 5:**
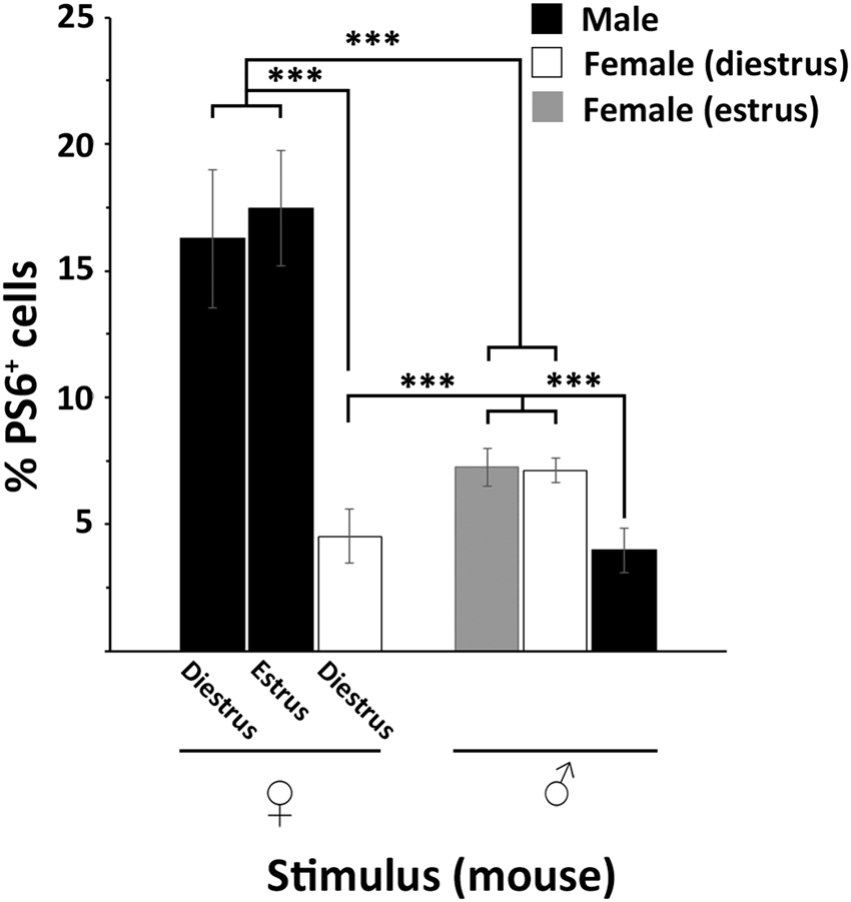
VNO responses in mice challenged with conspecifics. Female and male mice are paired to a conspecific and the percentage of pS6 positive cells is evaluated in the VNO. pS6 immunoreactivity in female is analysed during the oestrus and dioestrous phase. Males respond to oestrous and dioestrous females with a significantly higher number of pS6 immunoreactive cells than the opposite condition (oestrous and dioestrous females versus male stimulus; dioestrous females, F = 8.08, p = 0.001; oestrous females, F = 4.34, p < 0.001). Females and males respond with a lower number of pS6 immunoreactive cells in the VNO to the chemical cues emitted by the same-sex animal than to the opposite-sex one (male, F = 0.59, p < 0.001, dioestrous female, F = 3.01, p < 0.001). No effect of the oestrous phase is reported (n = 7 for each group; means ± s.d.).

We also studied the pS6 expression in a more physiological and restricted context. Therefore, in a series of experiments, we paired an isolated CD1 mouse with a Balb/c mouse of the opposite sex for an hour in a neutral cage containing fresh bedding. The experiment was performed taking into account the oestrous phase (dioestrous and oestrus) of both stimulated and stimulating females (Fig. 5). We found that the oestrous of the stimulating mouse, compared to dioestrous, did not modify the number of pS6 immunoreactive cells in the VNO of male mice. Similarly, pS6 expression in the VNO of females stimulated by a male was not influenced by the oestrous cycle. However, in all cases, as observed for the soiled bedding, the VNO of males responded to oestrous and dioestrous females with a significantly higher number of pS6 immunoreactive cells than the opposite condition (dioestrous females, F = 8.08, p = 0.001; oestrous females, F = 4.34, p < 0.001). We have shown above that male or female bedding activated a much lower number of cells in the VNO of the same sex of the stimulus than of the opposite sex, suggesting a desensitization mechanism due to the natural self-exposure to pheromones. Here, however, experimental mice were caged in relatively dense groups, making it difficult to determine whether adaptation occurred in the presence of cage-mate scent or also self-odours. Thus, isolated CD1 mice were also paired with animals of the same sex. Our data show that female and male VNO responded with a lower number of pS6 immunoreactive cells to the chemical cues emitted by the same-sex animal than to the opposite-sex ones (male as a stimulus, F = 0.59, p < 0.001, dioestrous female as a stimulus F = 3.01, p < 0.001). This last result seems to confirm that adapting mechanisms to pheromonal stimuli of the same sex are also displayed in a physiological context.

## Discussion

In this work, we have studied the response of vomeronasal neurons of male and female mice to a large array of pheromonal stimuli (soiled beddings or encounters with conspecifics) via the immunological detection of the S6 ribosomal protein phosphorylation. We found that this method of analysing the neuronal activity of the vomeronasal organ in living animals is fast and more sensitive than the immunological detection of other neuronal markers reported in literature (c-Fos, Egr 1, for example)^28,29,35^. This method has been employed in detecting the neuronal activity in the central nervous system and in the olfactory system. Very recently, Tsunoda and colleagues reported pS6 phosphorylation of VSNs activity in response to rat tear stimulation^32^. In this report, we have confronted the activation in VSNs of female and male CD1 mice in response to intraspecific and heterospecific stimuli. In our hands, male VNO responded to female stimuli with a higher density of pS6-positive neurons than female VNO to male stimuli, confirming the observations made by He and colleagues in VNO slices of transgenic mice expressing the calcium indicator G-CaMP2, stimulated with urinary cues^36^. This result may reflect a more complex pheromonal blend constituting female excretions. We were able to confirm these data with stimuli from all mouse strains that we have tested, although both female and male bedding collected from the C57/Bl6 strain retained a slightly lower pheromonal activity than the other strains. Interestingly, this greater sensitivity of the male VNO in detecting female cues than the opposite condition is completely abolished if animals were not allowed to physically contact stimuli, suggesting that a significant proportion of female pheromonal molecules are chemically non-volatile. This finding seems to support the results reported by Nodari and colleagues, who showed that sulphated steroids, a group of barely volatile molecules which are specifically contained in female urine, account for a large fraction of the electrical activity triggered by this stimulus^17^. Sulphated steroids have been reported to selectively bind specific classes of V1Rs but not V2Rs^12,13,37^. Consistent with this observation, we found that female cues activate a large population of neurons mainly located to the apical layer of male VNO that showed a low co-expression with V2Rs. In contrast, male scent seems to activate neurons throughout the sensory VNO.

Intriguingly, and in contrast to previous physiological studies based on electrophysiological recordings and Ca-imaging readouts^17,20,36^, we found that the VNO of both male and female mice responds to the same sex cues with lower neuronal activation than to the opposite sex cues. This observation is also confirmed when the source of the stimulus was the living animal rather than the soiled bedding. According to the consensus, mouse pheromone receptor genes do not show an evident sexual dimorphism based on both sequence and expression patterns^6^. Hence, and in contrast to our data, it would be expected that a given male or female stimulus activates the same number of chemosensory neurons in both male and female VNO. Thus, our results suggest the existence of a filtering mechanism to adapt the VNO to a persistent stimulation with pheromonal cues originating from the same animal or from animals of the same or opposite sex or from heterospecifics. This is also confirmed by experiments performed on castrated male mice. These mice do not produce most masculine pheromones and therefore they respond to intact male cues with a much stronger activation of the vomeronasal neurons, being less adapted to these stimuli. In contrast to the main olfactory system, where desensitization of chemosensory neurons upon odorant stimulation is unequivocally demonstrated^24,38^, adaptive mechanisms in the vomeronasal neurons are indeed an open question. Spehr and colleagues provided evidence that pheromone-evoked field potentials in intact VNO and in individual voltage-clamped VSNs undergo sensory adaptation, manifested by a time-dependent decline in sensitivity during prolonged or repetitive stimulation^25^. They also demonstrated that the complex Ca^++^-Calmodulin is responsible for this effect by inhibition of the diacylglycerol activated TrpC2 current^25^. However, other electrophysiological studies also failed to observe VSN adaptation when individual stimuli were delivered over a period of several hours^17,20,36^. Our *in-vivo* results show that pS6 immunoreactivity, employed as a marker of neuronal activity^30,39^, returns to background level in animals that are exposed to prolonged stimulation. However, electrophysiological adaptation occurs with a short latency after stimulus application, whereas the complete dephosphorylation of the S6 protein by a prolonged stimulation is a process that lasts several hours before it is fully accomplished. Indeed, after the removal of a short stimulus, S6 protein phosphorylation displays a similar time course. Similarly, several hours are required before VSNs, adapted to a given stimulus, respond to the same stimulus by S6 protein phosphorylation. This evidence suggests that biochemical and electrophysiological adaptation differs in terms of a temporal profile. Indeed, it remains possible that in our *in-vivo* experimental conditions, given the high sensitivity of the VNO to picomolar concentration of pheromones^11,40^, the temporal scale of the desentisization/resensitization process is dramatically amplified by the fact that the complete removal of the stimulus might be difficult to accomplish (odours may stick to the body after stimulus retrieval for hours). Electrophysiological measurements are exquisitely reliable in determining neuronal activation; however, *ex vivo* experiments require organ removal and extensive washing and conditioning in physiological solutions. Thus, it is possible that resensitization of the VSNs may occur much faster than observed in the *in-vivo* experiments, here reported.

Nevertheless, it should be considered that, although it is likely that S6 protein phosphorylation, as for the olfactory system^31^, reflects neuronal activation in the VNO, we largely ignore the adaptive mechanisms here reported, their potential role and to what extent the induction of pS6 is related to electrophysiological changes. In recent work, von der Weit and colleagues^41^ demonstrated that odour stimulation induces a reversible decrease in the transcription of genes corresponding to activated receptors. It is then possible that S6 phosphorylation may also be correlated to the mechanisms underlying this effect. Interestingly, the temporal scale of the RNA downregulation in the olfactory neurons appears approximately similar to the sensitization/desensitization effects that we observe in the vomeronasal neurons.

In this context, it is interestingly to note that the chemosensory neurons of the Grueneberg ganglion, a peripheral olfactory organ that respond to stress odorants, express vomeronasal receptors and seem to undergo adaptation to c-Fos expression following a prolonged exposure to stimuli^42,43^.

In this work, we have also tested heterospecific stimuli and, in contrast to intraspecific cues, observed no difference in the number of pS6 immunoreactive neurons between sexes. This is in line with that which was reported in a previous *in vivo* study employing a riboprobe to detect the expression of the proto-oncogene Egr 1^12^. In our study, we found that the rat bedding mix (female and male) represents a very strong stimulus for the mouse VNO as up to 30% of the VSNs showed activation. However, when mice were exposed to female and male rat bedding, respectively, we identified a distribution of the pS6 immunoreactive cells that reflects the situation observed for mouse stimuli. In fact, female rat stimuli clearly activate apical neurons, whereas male stimuli activate neurons throughout the VNO. A similar pattern of expression is also observed with hamster stimuli, although they are generally less effective in activating VNO neurons (not shown). Interestingly, male mice adapted to female mouse scent showed a 30% reduction of response to female rat stimuli, suggesting that a significant proportion of VSNs that respond to mouse also respond to rat cues. This may indicate the presence of classes of pheromonal molecules that are shared between species for social communication. In line with our observation, Tsunoda and colleagues^32^ have recently demonstrated that a rat lacrimal protein, namely CRP1, activates VSNs of both rats and mice affecting specific behavioural performances. According to these observations, in future works, it will be interesting to investigate if the modality of communication across species may be extended to individuals belonging to different animal orders including humans.

## Materials and Methods

### Animals

Mice (*Mus musculus*, CD1 strains) were housed under a 12 h light/dark cycle. All experiments were carried out between 9:00 a.m. and 12:00 a.m.

B6;129P2-*Omp*^*tm3Mom*^/MomJ mice in which the coding region and part of the 3′ non-translated region of the *Omp* gene was replaced by GFP were from Jackson Laboratory and only used to confirm pS6 immunoreactivity in mature vomeronasal neurons (Supplementary Fig. S2).

Three-four week old CD1 male mice were castrated and utilized for experiments after one month from surgery.

### Stimuli and experimental procedure

Bedding samples from mice (CD1, C57/Bl6 and Balb/c strains), rat (*Rattus norvegicus*, reared wild type) and hamster (*Mesocricetus auratus*) were freshly collected from at least three cages and pooled before each experimental session.

Fox and bobcat urine were purchased from PredatorPee Store and sprayed over clean bedding before the experiments.

Freshly dead snakes (*Natrix natrix*) were collected from the roads and the skin removed and cut into pieces.

For conspecific stimuli, bedding samples were collected from CD1, Balb/c and C57/Bl6 strains. Since pheromone production is strongly influenced by the sexual and social status of the animals (oestrus cycle and dominance) which varies over time, we sampled materials from cages housing several animals for over 5–7 days. Bedding, when necessary, was stored at −80 °C. When bedding stimuli were used, these were added to a clean cage just before introducing the animals. Single colonies of subject animals (sexually naïve CD1 male or female mice) were moved from the animal house and isolated for 3–4 days in a clean room before each experiment. During the experimental session with soiled bedding, an individual animal was introduced in a cage containing the stimulus and left for 1 hr unless indicated in the text. Control experiments were conducted using clean bedding under identical conditions.

In behavioural experiments where CD1 mice were paired before VNO collection and processing, animals of the same sex were singly housed for 3 days in the same clean room under a fume hood to minimize the contact with volatiles molecules. Mice for pairing were then introduced, at the same time, in a cage containing clean bedding. Animals were allowed to investigate each other for 1 hr prior to sacrifice. Using this procedure, male mice could be paired without displaying aggressive behaviour.

Mice were sacrificed by cervical dislocation and the VNO was then dissected, fixed for 4 hrs at 4 °C with a solution of 4% phosphate-buffered paraformaldehyde, and cryoprotected overnight at 4 °C in 30% sucrose. Subsequently, tissues were included in OCT embedding solution (CellPath, UK) and frozen in liquid nitrogen -cooled pentane.

All animal protocols complied with the ethical guidelines for the care and use of laboratory animals issued by the Italian Government and were approved by the Animal Welfare Committee (OPBA, Organizzazione Per il Benessere Animale) of the University of Parma. All methods were carried out in accordance with the relevant guidelines and regulations.

### Immunohistochemistry

Cryostat-cut sections (20 μm) were blocked in 1% albumin, 0.3% Triton X-100 for 20 min and incubated with the anti-pS6 antibody (Ser240/244, Cell Signalling Technology, 1: 250 dilution) in the same blocking solution for 40 hrs at 4 °C. Sections were revealed with an anti-rabbit IgG conjugated with Alexa488 (or Alexa568) (1:350) (Invitrogen).

For double label immunohistochemistry, after pS6 development, sections received treatment with 3% normal rabbit serum for 1 hr at room temperature and were then incubated with the biotinylated anti-V2R (panC or panD) antibody^34,44^ (used at 1: 100 dilution) in the presence of 1% normal rabbit serum for 24 hrs at 4 °C. The immune-complexes were visualised with streptavidin-Alexa488 (or streptavidin-Alexa568) (1: 350).

Fluorescent images were obtained using a Zeiss LSM 510 Meta System scan integrated with Axiovert 200 M inverted microscope (Carl Zeiss, Jena, Germany).

### Cell Counts and Statistical analysis

Cryostat cut, coronal VNO sections to be analysed were collected in the central part of the VNO to obtain a better definition of the boundaries between the apical and basal layers of the neuroepithelium. Immunolabelled sections were counterstained with DAPI to display all nuclei. To count cells, sections were analysed with a 25× objective and images were processed with the Photoshop CS6 Extended program. A Nikon DS-2Mv Camera was used and the exposure time was set at 300 msec for images subjected to cell counting. The number of positive cells was calculated per selected area and as a percentage of total VNO cells. Given the very low background of the anti-pS6 antibody (see Supplementary Fig. S1), a cell was deemed positive when it was distinguishable from background.

Total VNO cells were identified by incubating sections for 10′ in a PBS solution of DAPI (1: 10,000). Eight to 10 sections per animal were normally counted. Five to 10 animals for each experimental point were used. Mice living in three different colonies composed each experimental group. The experimenter who counted sections (corresponding author) was blinded to the experimental conditions (except for the experiment in Supplementary Fig. S2).

Sample size was determined to the conventional practice for behavioural tests. All data are expressed as means ± s.d. Two-way ANOVA t-test followed by Bonferroni post-hoc test was used for evaluation of immunostaining.

### Data availability statement

The datasets generated and analyzed during the current study are available from the corresponding author.

## Electronic supplementary material

Supplementary Figures
