# Supplementary material for: In-vivo activation of vomeronasal neurons shows adaptive responses to pheromonal stimuli
Source: Sci Rep. 2018 May 31;8:8490. doi: 10.1038/s41598-018-26831-5 (PMC5981476; doi:10.1038/s41598-018-26831-5)
Supplement: Supplementary file 1 — Supplementary Figures [file 41598_2018_26831_MOESM1_ESM.pdf]

In-vivo activation of vomeronasal neurons shows adaptive responses to pheromonal stimuli

Lucia Silvotti, Rosa Maria Cavaliere, Silvana Belletti and Roberto Tirindelli*

Department of Medicine, Neuroscience Unit, University of Parma, 43125, Parma, Italy.

*, corresponding author, robertin@unipr.it

Supplementary information

SUPPLEMENTARY FIGURE S1

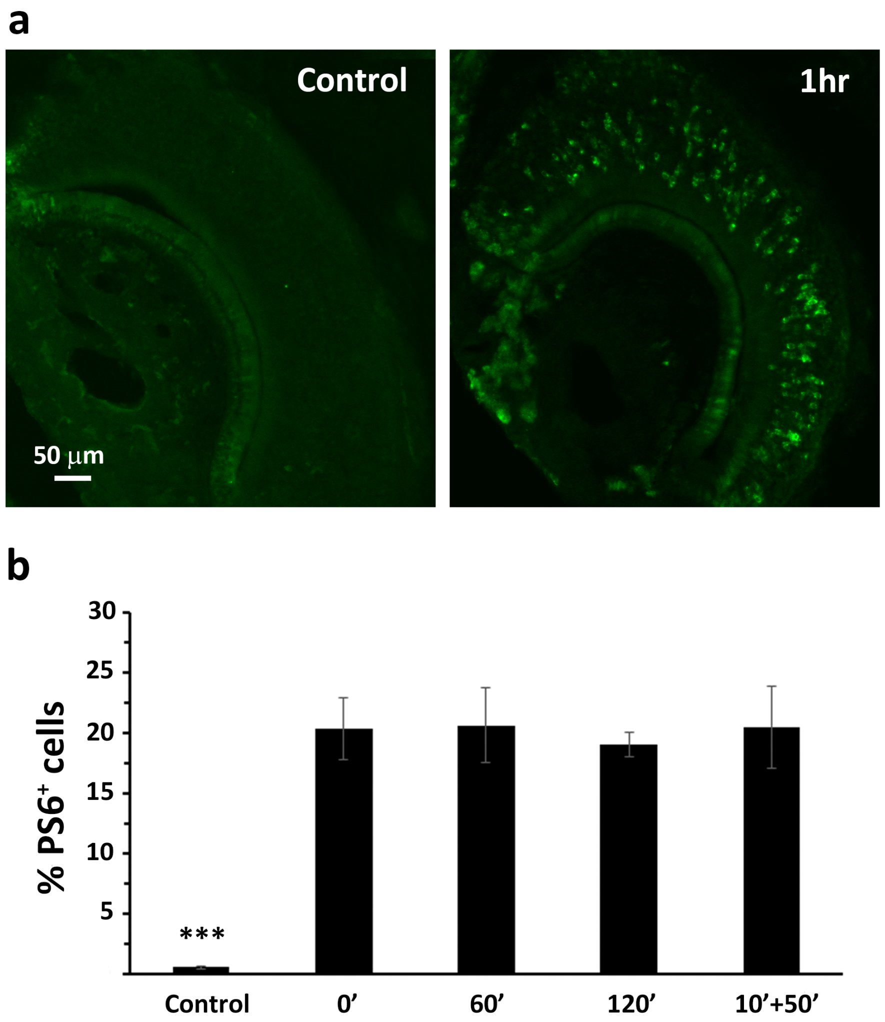

Supplementary Figure S1. Optimisation of the stimulus protocol. **(a)** VNO of CD1 male mice exposed to Balb/c male and female bedding or clean bedding (control) for 1 hr before sacrifice. **(b)** pS6 immunoreactivity in the VNO of CD1 male mice exposed: to clean bedding for 60' before sacrifice (control); to a bedding mix of male and female Balb/c mice for 1hr and then sacrificed at 0', 60' and 120' after stimulus removal (bars 2-4); to bedding mix for 10' and then transferred to a clean cage with fresh bedding for 50' before sacrifice (bar 5). The number of pS6 positive neurons remains constant after 2hrs from stimulus removal. Ten-minute exposure to stimulus was sufficient to elicit a nearly full response in the VNO ($n = 5$ for each group; means ± s.d.; ***, $p < 0.001$).

SUPPLEMENTARY FIGURE S2

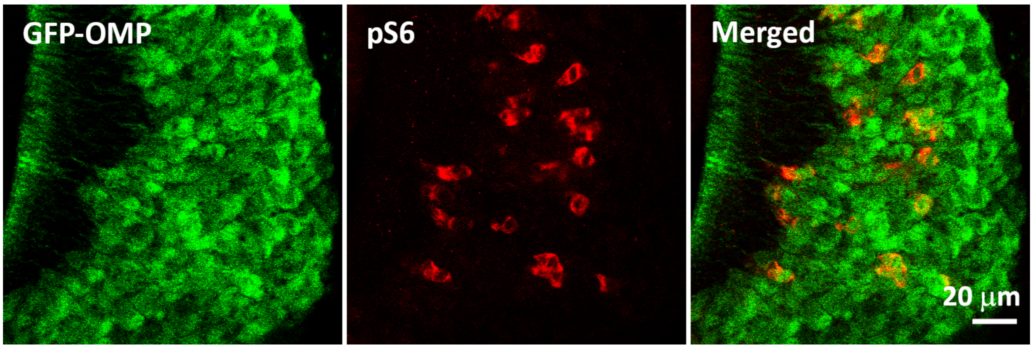

Supplementary Figure S2. Co-localization between pS6 and OMP-GFP. pS6 immunoreactivity in the VNO of OMP-GFP male mice exposed to a bedding mix of male and female Balb/c mice. The majority of pS6 positive neurons are also positive for OMP.

SUPPLEMENTARY FIGURE S3

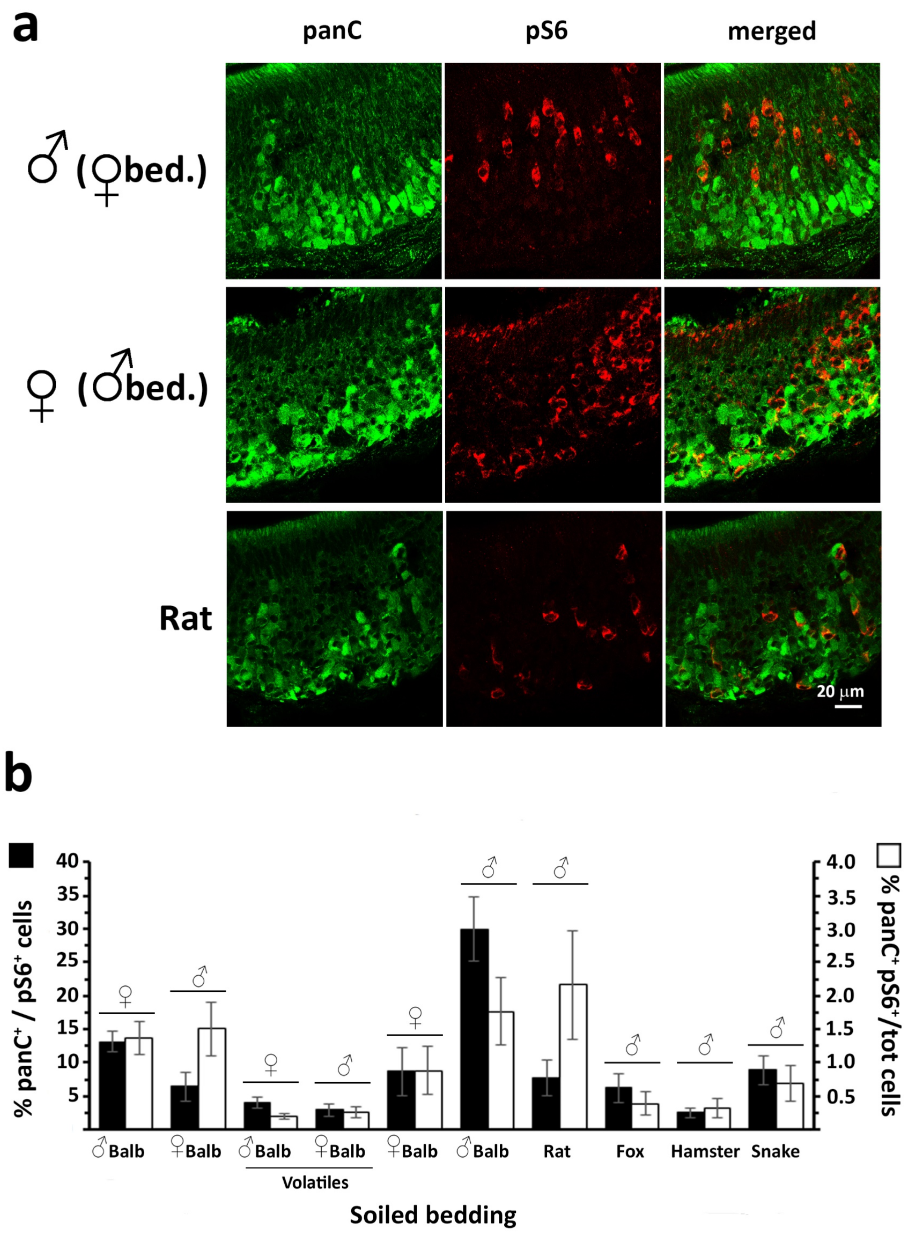

Supplementary Figure S3. Localization of pS6 in V2R expressing neurons following conspecific and heterospecific stimuli. **(a)** Double label immunohistochemistry with an antibody, which recognises all V2R-expressing neurons (panC) in mice, exposed to conspecific and heterospecific (female and male rat bedding mix) stimuli. **(b)** Indicated is the percentage of V2R positive neurons expressing pS6 (black bars and left ordinate) and the percentage of neurons co-expressing pS6 and V2Rs over the total VSNs in a selected area (white bars and right ordinate). Male cues show a significantly higher percentage of V2R/pS6 co-expression than female cues but this difference is abolished when mice are exposed to volatile odours, only. For heterospecific stimuli, a mix of male and female bedding is used (n = 7 for each group; means ± s.d.).

SUPPLEMENTARY FIGURE S4

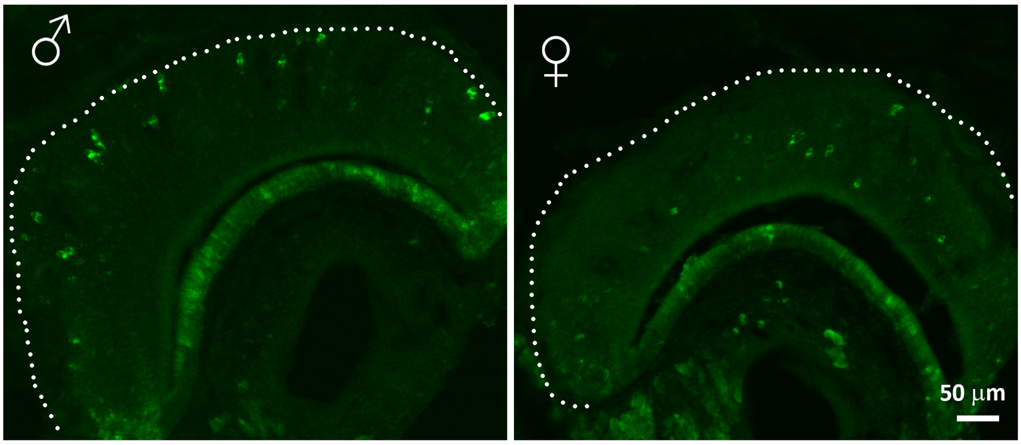

Supplementary Figure S4. pS6 immunoreactivity in response to stimuli of the same sex. CD1 male and female mice are exposed to Balb/c bedding of the same sex. Note the low density and the different spatial distribution of labelled neurons.

SUPPLEMENTARY FIGURE S5

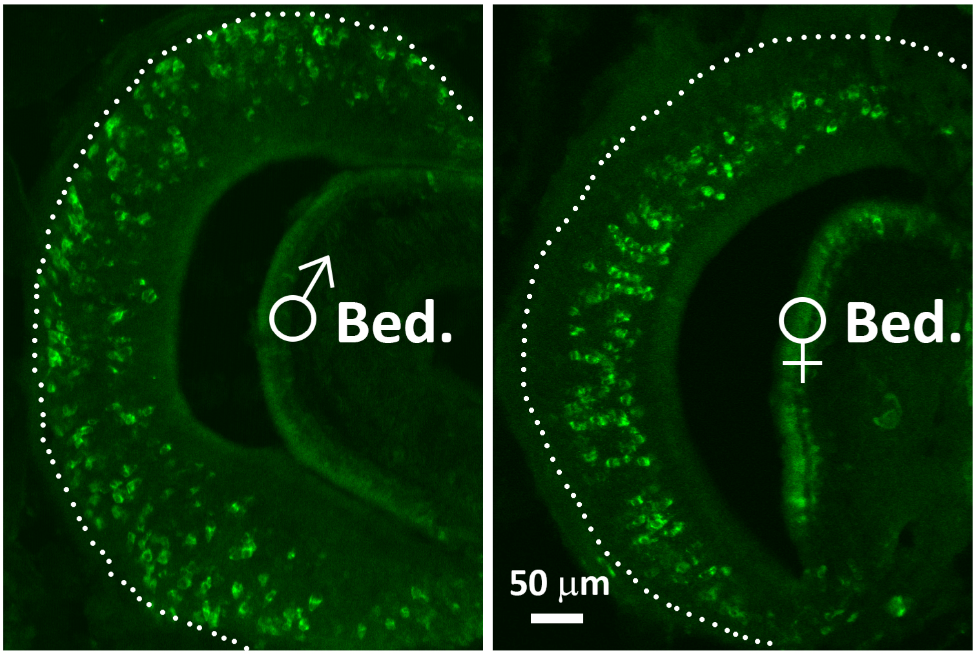

Supplementary Figure S5. Male and female heterospecific stimuli activate different neuronal subpopulations. pS6 immunoreactivity in the VNO of CD1 male mice exposed to bedding collected from male and female rats, respectively. Note the different expression pattern of pS6 in the two VNO sections.

SUPPLEMENTARY FIGURE S6

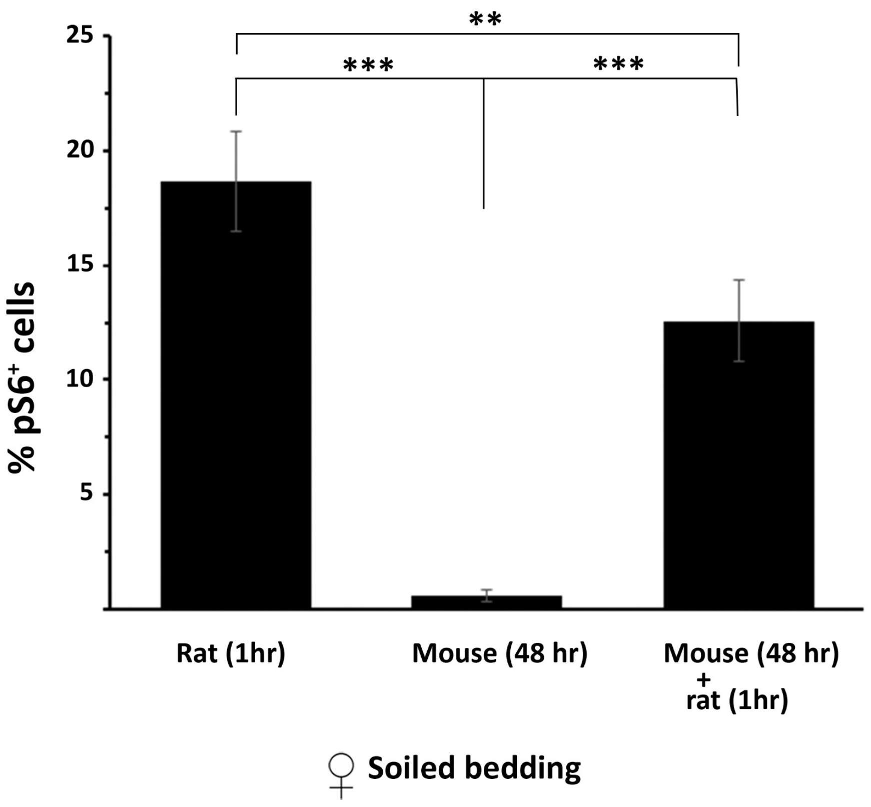

Supplementary Figure S6. VNO of male mice adapted to female cues shows a reduced sensitivity to rat pheromones. pS6 immunoreactivity in the VNO of CD1 male mice exposed to rat bedding before and after adaptation with mouse female bedding for 48 hrs ($n = 7$ for each group, means ± s.d.; ***, $p < 0.001$; **, $p < 0.01$).

SUPPLEMENTARY FIGURE S7

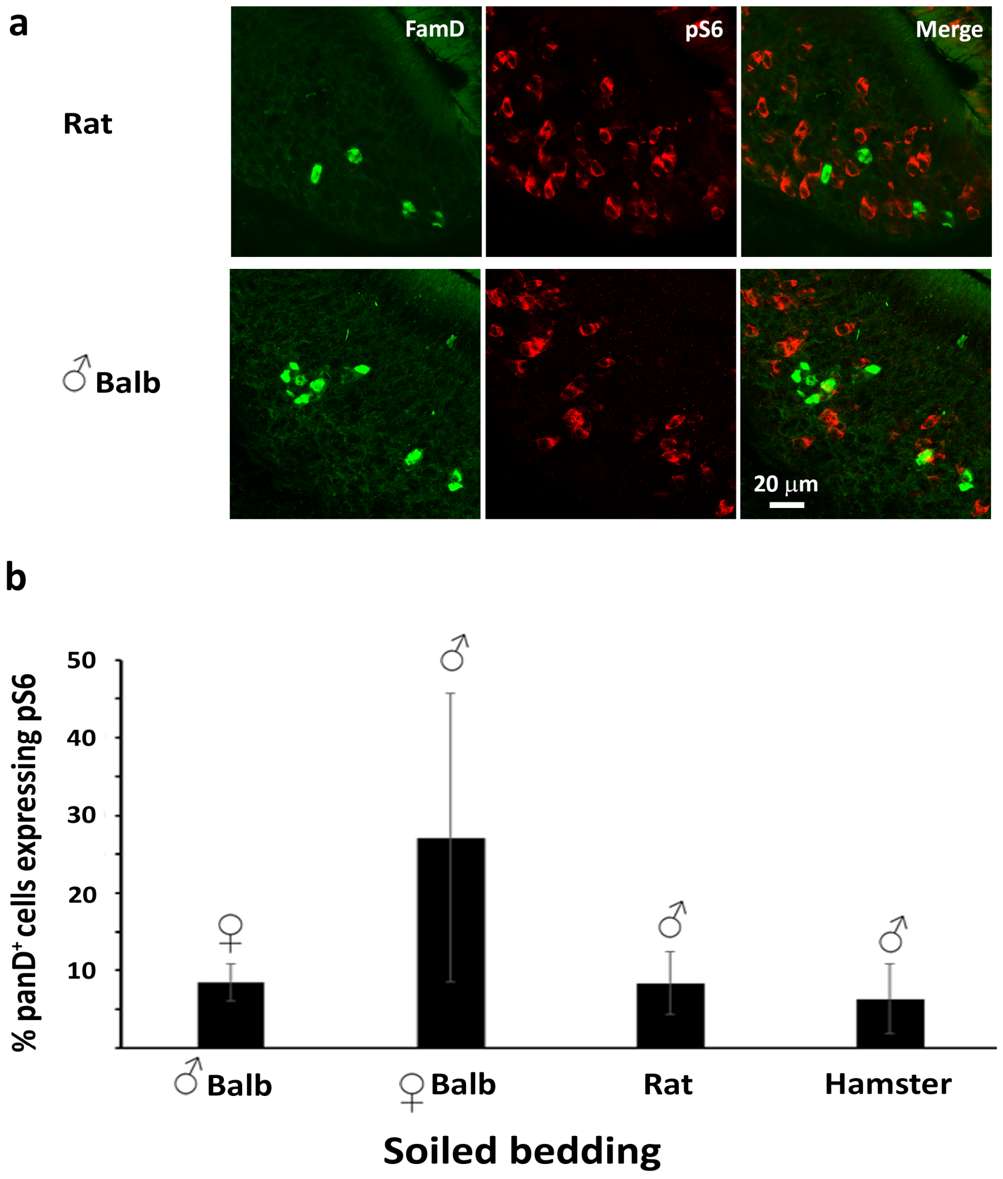

Supplementary Figure S7. Activation of V2R family-D expressing neurons upon stimulation with conspecific and heterospecific cues. **(a)** Double label immunohistochemistry with anti-pS6 and with an antibody recognising all family-D V2Rs in a VNO of male mouse exposed to rat or Balb/c mouse bedding (male + female). Note the low level of co-expression. **(b)** Histograms showing the percentage of family-D expressing neurons that also express pS6 in mice stimulated with soiled bedding of heterospecific (male + female) and conspecific animals (n = 7 for each group; means ± s.d.).
